# Analysis of the gut microbiome in obese native Tibetan children living at different altitudes: A case–control study

**DOI:** 10.3389/fpubh.2022.963202

**Published:** 2022-11-24

**Authors:** Wenqi Du, Linxun Liu, Yan Ma, Qinfang Zhu, Ruhan Jia, Ying Han, Ziyi Wu, Xin Yan, Ainiwaer Ailizire, Wei Zhang

**Affiliations:** ^1^Research Center for High Altitude Medicine, Qinghai University School of Medicine, Xining, China; ^2^Department of Public Health, Qinghai University School of Medicine, Xining, China; ^3^Key Laboratory for Application of High Altitude Medicine in Qinghai Province, Qinghai University, Xining, China; ^4^General Surgery Department, Qinghai Provincial People's Hospital, Xining, China; ^5^Qinghai-Utah Joint Research Key Lab for High Altitude Medicine, Qinghai University School of Medicine, Xining, China

**Keywords:** obesity, high altitude, schoolchildren, gut microbiome, metabolism

## Abstract

**Objective:**

To explore the relationship between intestinal flora and obesity in Tibetan children at different altitudes.

**Methods:**

Using16S rRNA gene sequencing results and blood lipid metabolism indexes to study the characteristics of the intestinal flora present in faeces and changes in blood lipid metabolism in obese children in Tibet who reside at different altitudes and to study correlations between blood lipid metabolism indicators and the intestinal flora composition.

**Results:**

The results showed the following. (a) The triglyceride (TG) and low-density lipoprotein cholesterol (LDL-C) levels in the obesity groups were higher than those in the normal-weight groups, and those in the high-altitude obesity groups were lower than those in the low-altitude obesity groups. (b) The 16S rRNA gene sequencing results showed that altitude affected the composition and relative abundance of the gut microbiota. These parameters were basically the same among the low-altitude groups, while they were significantly lower in the high-altitude groups than in the low-altitude groups. (c) Groups that lived at different altitudes and had different body weights had different dominant bacterial genera. *Megamonas* was closely related to obesity, and its relative abundance in the low-altitude groups was higher than that in the high-altitude groups. *Prevotella* was associated with altitude, and its relative abundance in the high-altitude groups was higher than that in the low-altitude groups. In addition, *Prevotella* elicited changes in the abundance of Escherichia-Shigella. The lower prevalence of obesity and incidence of intestinal inflammation in those living at high altitudes were related to the abundance of *Prevotella*. (d) There were correlations between the gut microbiota composition and lipid metabolism indicators. The abundance of *Romboutsia* was positively correlated with TG and LDL-C levels but negatively correlated with high-density lipoprotein cholesterol (HDL-C) levels. The abundance of *Akkermansia* was negatively correlated with LDL-C levels, and the abundance of *Blautia* was negatively correlated with body mass index (BMI) and LDL-C levels.

**Conclusions:**

The intestinal flora diversity varied by body weight and altitude, with lower diversity in those at higher altitudes and with lower body weights. *Prevotella* likely plays a role in suppressing obesity at high altitudes.

## Introduction

Obesity is a global health problem. Obesity is a complex, multifactorial and largely preventable disease that affects approximately one-third of people worldwide (1). Obesity is linked to type 2 diabetes (2), respiratory disease (3), cardiovascular disease (4, 5), female infertility (6, 7), and nonalcoholic fatty liver disease (7), and it can also be a risk factor for cancer (8). With improvements in living standards and changes in diets, childhood obesity has become increasingly prevalent worldwide, and children are becoming affected by obesity at a younger age (9, 10). Jia et al. (11) found that the prevalence of overweight and obesity in 6- to 17-year-old children increased from 11.7 to 25.2% and from 2.8 to 10.1% from 1991 to 2011, respectively. Additionally, the prevalence of obesity in children and adolescents in China is rapidly increasing; it increased at a rate of 0.36% annually from 2000 to 2011 (12). Many studies have shown that obesity in childhood is associated with an increased risk of obesity in adulthood (13, 14). This correlation suggests that attention should be given to the problem of childhood obesity.

Obesity is related to not only the imbalance of energy intake but also genetics, the environment and lifestyle; however, the underlying causes of obesity are still unclear (15). Among the nongenetic factors associated with obesity, intestinal flora composition has been recognized as a regulator of obesity, and a correlation between changes in the intestinal flora composition and body weight has been observed in a number of studies conducted in animal models of obesity (16–18). However, the status of the intestinal microbiome in children with obesity has not been well studied. Several studies (19–21) have revealed that the gut microbiome composition might be a factor that affects obesity. Changes in the biological diversity of the intestinal flora can affect the health and growth of the host to a large extent, but the specific factor that regulates the gut microbiome remains poorly understood.

The Tibetan Plateau is the highest plateau in the world, with a mean elevation of 4,500 m (22). It is characterized by unique environmental conditions, such as low pressure, low temperatures, low humidity and high radiation (23). A high-altitude hypoxic environment causes disorder of the intestinal flora; hypoxia causes inflammation, and inflammation aggravates hypoxia in tissues. In a high-altitude hypoxic environment, the intestinal mucosal barrier is highly prone to damage. This damage causes the translocation of intestinal bacteria and toxins, which causes immune system activation and the release of inflammatory mediators, resulting in disruption of the intestinal flora composition. Some studies have shown that factors related to altitude and anoxic environments have significant effects on the composition of the gut microbiome (24), and high altitude may contribute to shaping the human gut microbiota composition (25). The study by Santos (26) showed that the rate of overweight/obesity in children who reside at high altitudes is lower than that in children who reside at low altitudes. The results of our research group's previous epidemiological survey showed that the prevalence of overweight in 1,561 children aged 7–12 years who resided on the Qinghai-Tibet Plateau was 8.5% (9.9% for boys and 7.1% for girls), and the prevalence of obesity was 6.3% (6.0% for boys and 6.5% for girls); these values were much lower than those observed in the rest of China (11). Therefore, we sought to examine whether altitude affects the gut microbiota composition and thus influences obesity.

## Materials and methods

### Ethics statement

The experimental protocol was established according to the ethical guidelines of the Declaration of Helsinki and approved by the ethics committee of Qing Hai Provincial People's Hospital, Xining, China.

### Study design

A cross-sectional study was designed to explore the association between high altitude residency and obesity with respect to lipid levels and gut microbiota diversity by comparing two indigenous communities who live at different altitudes. The study was approved by the Human Rights Committee of the University of Buenos Aires. Each parent provided written informed consent after the study was explained and before it was initiated.

### Participants

We enrolled 70 subjects in our research study. All the participants were Tibetan children aged 7–12 years with the same eating habits. Using simple random sampling, we selected one primary school as a research site in each altitude area. Based on the China Center for Disease Control and Prevention (CDC) growth charts (27), we divided the subjects into four groups: obese children (*N* = 15, high-altitude obese children, HOB) and normal-weight children (*N* = 20, high-altitude normal-weight children, HN) who lived at an altitude of 4,500 m in Ma Duo County (34°52′12″N, 98°15′36″E), Guoluo Tibetan Autonomous Prefecture, Qinghai–Tibet Plateau; and obese children (*N* = 15, low-altitude obese children, LOB) and normal-weight children (*N* = 20, low-altitude normal-weight children, LN) who lived at an altitude of 1,500 m in Hai Dong County (36°18′N, 102°48′E), Hai Dong city, Qinghai–Tibet Plateau. A detailed questionnaire that included questions about age, sex, parental education level, history of breastfeeding, and family history of obesity was administered.

### Inclusion and exclusion criteria

The inclusion criteria were as follows: (a) age 7–12 years old (28) and living in the research area for at least 5 years; (b) attendance at the selected boarding school; and (c) a diet containing grains, beef, and fresh vegetables. The exclusion criteria were as follows: (a) use of antibiotics or probiotics within the past 4 weeks; (b) gastrointestinal diseases (e.g., diarrhea) within the past 4 weeks; (c) frequent constipation; or (d) failure to agree to participate in this study. All the enrolled subjects were healthy, with no history of gastrointestinal disease, liver disease, hypertension, or diabetes, as demonstrated by their medical histories and physical examinations.

### Anthropometric measurements

Height, weight, waist circumference and hip circumference were measured according to the protocol of the International Society for the Advancement of Kinanthropometry (29). Height was measured to the nearest 0.1 cm while the child stood upright against a mounted stadiometer with bare feet. In addition to height, waist and hip circumferences were measured. A digital weighing scale (Huawei3Pro, ShengZhen, China) that was calibrated regularly to the nearest 0.1 kg (after every 10 measurements) was used to measure body mass. We asked children to wear only a t-shirt and pants. Based on the height and body mass measurements, body mass index [BMI (weight/height^2^)] was calculated using the following formula:


$$\begin{array}{l} BMI=masskg/{\left[height\;\left(m\right)\right]}^{2} \end{array}$$

### Blood sample collection and analysis

Venous blood samples (5 ml) were collected by venepuncture into Vacutainer tubes, and plasma levels of glucose (GLU), triglycerides (TGs), total cholesterol (TC), high-density lipoprotein cholesterol (HDL-C) and low-density lipoprotein cholesterol (LDL-C) were analyzed with an LW-C400 automatic biochemical analyser (Landwind, Shenzhen, China).

### Stool samples

Using sterile fecal boxes, fresh fecal samples (5 g) were collected from obese and control children within 2 h of defecation. Fecal samples were quickly placed in an ultralow temperature freezer (−80°C) for further processing and testing.

### Determination of SCFAs contents in feces

Fresh fecal samples (2 g) were mixed with 3 ml deionized water and homogenized. The mixture was left for 20 min at room temperature and centrifuged at 14,000 rpm for 15 min. Then, the supernatant was transferred to new EP tubes. Then, 3 ml deionized water was added to the precipitate, and all of the above operations were repeated again. The extraction was repeated twice, and the supernatants were combined. Next, it was filtered through a 0.22 μm filter. A 1 ml sample solution was injected into the GC system. Chromatographic analysis of fecal samples was performed using ISQ Single Quadrupole GC–MSMS (Thermo Fisher Scientific), Trace 1300 Series Gas Chromatograph (Thermo Fisher Scientific) and TG-5MS Separation column (30 m × 0.32 mm, 0.25 μm). The injection port temperature was 160°C. The GC oven was programmed with an increasing starting temperature from 50°C for 1 min to 220°C with 10°C/min for 5 min. A volume of 0.2 μl of the sample was injected. Electron impact ionization (EI) was used as an ionization source for the GC/MS analysis at 70 eV. The injection temperature was 230°C, and the transfer line and ion source were set to 250°C. XcaliburTM software (Thermo Fisher Scientific) was used for the automatisation of the GC–MS system and for data acquisition. Sample quantification was obtained by means of acetic, propionic, and butyric acid standard curves (Sinopharm). Construction of Standard Curve The standard calibration curves were constructed using seven concentrations: 9.325, 18.75, 37.5, 75, 150, 300, and 900 μg/ml. The concentration of each sample was obtained by the standard curve. The standard curves of acetic acid, propionic acid, and butyric acid were *Y* = 3.796e5*X* + 9.363e4, *Y* = 3.936e5*X* + 4.003e5, and *Y* = 1.366e6*X* + 1.151e6, respectively (30, 31).

### DNA extraction

Total genomic DNA was extracted from the samples using the acetyltrimethylammonium bromide (CTAB) method (28) DNA concentration and purity were analyzed using 1% agarose gels. According to the concentration, the DNA samples were diluted to 1 ng/μl using sterile water. An equal volume of 1 × loading buffer (containing SYBR green) was mixed with the PCR products, and electrophoresis was performed on a 2% agarose gel to visualize the PCR products. PCR products were mixed in equidensity ratios. Then, the PCR products were purified with a Qiagen Gel Extraction Kit (Qiagen, Germany). The DNA purity and concentration were analyzed by measuring the optical density (OD) at wavelengths of 260 and 280 nm with a NanoPhotometer^®^ spectrophotometer (Implen, Munich, Germany) and then calculating the OD260:OD280 ratio. The DNA concentrations were measured with the Qubit^®^ dsDNA Assay Kit in a Qubit^®^ 2.0 Fluorometer (Life Technologies, Camarillo, CA, United States).

### Metagenomic bacterial 16S rRNA gene sequencing assay

Based on many previous studies (32–34), we selected the V3–V4 region to study the microbiome through second-generation sequencing. 16S rRNA gene sequencing was performed by Novogene Bioinformatics Technology Co., Ltd., China. In brief, DNA samples were diluted to a concentration of 1 ng/μl in sterile water and then PCR amplified with the 515F/806R primer set (515F: 5′-GTGCCAGCMGCCGCGGTAA-3′, 806R: 5′-XXXXXXGG ACTACHVGGGTATCTAAT-3′). Sequencing libraries were generated using the TruSeq^®^ DNA PCR-Free Sample Preparation Kit (Illumina, USA) following the manufacturer's recommendations, and index codes were added. Library quality was assessed with a Qubit@ 2.0 Fluorometer (Thermo Scientific) and Agilent Bioanalyzer 2100 system. Finally, the library was sequenced on the Illumina NovaSeq platform, and 250-bp paired-end reads were generated.

### Data analysis

Paired-end reads were assigned to samples based on their unique barcodes and were truncated by removing the barcode and primer sequences. Paired-end reads were merged using FLASH (V1.2.7) (35), which is a very fast and accurate analysis tool that was designed to merge paired-end reads when at least some of the reads overlapped with the read generated at the opposite end of the same DNA fragment. The splicing sequences are called raw tags. Quality filtering of raw tags was performed under specific filtering conditions to obtain high-quality clean tags (36) according to the QIIME (V1.9.1) (37) quality control process. The tags were compared with those in the reference database (Silva138 database) using the UCHIME (28) algorithm to detect chimeric sequences, and the chimeric sequences were removed (38). Then, effective tags were finally obtained.

The data were analyzed and compared using SPSS version 27.0 (Chicago, Illinois, USA) and R software (v 2.15.3). Data are summarized as the mean (standard deviation, SD)/median (interquartile range, IQR) for continuous variables depending on normality. Student's t test was used to analyse significant differences in age, sex, height, weight, waist circumference, hip circumference, BMI and lipid levels among the schoolchildren who lived at different altitudes. Analysis of variance (ANOVA) was used to compare the content of short-chain fatty acids (SCFAs) in feces among the four groups. Significant differences in categorical variables are expressed as numbers and percentages. The chi-square test was used to analyse count data. The Kruskal–Wallis test was used to investigate significant differences in operational taxonomic units (OTUs) and the abundance-based coverage estimator (ACE), Chao 1, Simpson's, and Shannon's indexes among the four groups. To correct for type I errors, we applied the Bonferroni method for multiple comparisons between two groups. Differences in microbial community abundances between the obesity group and the control group were analyzed using the Wilcoxon rank sum test, and the significance of these differences was assessed using the false discovery rate (FDR). Principal coordinate analysis (PCoA) was performed using the WGCNA package, stat packages and ggplot2 package in R software (v 2.15.3) to compare the similarity of community structures. Multiresponse permutation procedures (MRPPs) (39) were used to analyse differences in the microbial community structure between groups. Linear discriminant analysis effect size (LEfSe) was performed to identify particular taxa with significantly different abundances between the two groups. An unweighted pair-group method with arithmetic means (UPGMA) clustering was performed as a type of hierarchical clustering method to interpret the distance matrix using average linkage by QIIME (2 v2022.2) software. Correlations between blood lipid levels and intestinal flora diversity were analyzed by Spearman's correlation analysis. *p* ≤ 0.05 indicated statistical significance.

## Results

### Basic participant information and serum biochemical indexes

A total of 70 subjects were included in the high-altitude groups (*n* = 35, normal/obese = 20/15) and low-altitude groups (*n* = 35, normal/obese = 20/15; Table 1). There was no significant difference in age, sex or height (*p* > 0.05; Table 1). Body weight, BMI, waist circumference and hip circumference were significantly different between the obesity group and the control group from the same altitude (*p* < 0.05; Table 1). There was also a significant difference between the obese groups from different altitudes (*p* < 0.05, Table 1). TG levels were significantly different between the obesity group and the control group from different altitudes (*p* < 0.05, Table 1), and LDL-C levels were significantly different between the obese groups from different altitudes (*p* < 0.05, Table 1).

**Table 1:** Characteristics of the participants by altitude.

**Characteristics**	**1,500 m**		***p*-value**	**4,500 m**		***p*-value**
	**LN (*n* = 20)**	**LOB (*n* = 15)**		**HN (*n* = 20)**	**HOB (*n* = 15)**	
Age	9.20 ± 1.44	8.20 ± 1.24	0.094	8.70 ± 1.53	9.53 ± 1.06	0.079
Male	11 (55.0)	8 (53.3)	0.922	8 (40.0)	8 (53.3)	0.433
Female	9 (45.0)	7 (46.7)		12 (60.0)	7 (46.7)	
Hight (cm)	135.6 ± 7.4	137.8 ± 9.7	0.450	135.8 ± 7.6	137.1 ± 6.5	0.618
Weight (kg)	25.8 ± 4.2	38.9 ± 5.0	0.000	24.9 ± 4.1	35.0 ± 4.5^*^	0.000
BMI (kg/m^2^)	13.9 ± 1.1	20.5 ± 2.2	0.000	13.5 ± 1.4	18.6 ± 1.8^*^	0.000
Waist (cm)	51.9 ± 4.6	70.5 ± 5.3	0.000	48.9 ± 5.4	61.6 ± 4.9^*^	0.000
Hip (cm)	57.3 ± 5.3	77.3 ± 5.1	0.000	55.0 ± 6.7	68.5 ± 4.9^*^	0.000
TC	3.9 ± 0.7	4.3 ± 0.9	0.116	4.5 ± 0.8	4.4 ± 0.5	0.814
TG	1.2 ± 0.3	1.6 ± 0.4	0.012	1.1 ± 0.4	1.4 ± 0.5	0.024
HDL	1.4 ± 0.9	1.1 ± 0.3	0.160	1.4 ± 0.3	1.1 ± 0.4	0.081
LDL	2.1 ± 0.4	2.3 ± 0.6	0.152	2.5 ± 0.6	2.8 ± 0.4^*^	0.068
GLU	4.8 ± 0.5	4.6 ± 0.5	0.224	4.8 ± 0.5	4.4 ± 0.9	0.122

LN means low-altitude obese children, LOB means low-altitude obese children, HN means high-altitude normal weight children, HOB means high-altitude obese children.

* Compared with LOB group (p < 0.05).

### High 16S rRNA gene sequence data preprocessing and quality control

A total of 6,130,974 high-quality and classifiable raw reads were obtained from 70 samples. We retained 5,991,598 clean reads after removing the low-quality sequences. A total of 3,109,972 and 2,881,626 clean reads were obtained for the high-altitude groups and the low-altitude groups, respectively. Clustering analysis assigned the microbial sequences to the same OTU if the samples had at least 97% similarity. We obtained 3,843 OTUs by high-throughput sequencing. These reads were classified into 43 phyla, 101 classes, 226 orders, 331 families, 576 genera, and 340 species. The rarefaction curves showed that our sequencing depth was sufficient (Supplementary Figure S1).

### α-Diversity analysis

α-diversity analysis was performed using four indexes, namely, OTUs and the Shannon index, Simpson index, Chao 1 index, and ACE index. There were highly significant differences in all four indexes among the four groups according to the Kruskal–Wallis test (*p* < 0.05; Table 2). The Shannon and Simpson indexes were significantly different in the different BMI groups from the same altitude according to the Bonferroni multiple comparisons test (*p* < 0.05; Table 2). The OTUs and Chao 1 and ACE indexes were significantly different between groups with the same BMI who were from different altitudes according to the Bonferroni multiple comparisons test (*p* < 0.05; Table 2).

**Table 2:** Fecal microbial HiSeq sequencing data.

**Group**	**OTUs**	**Shannon**	**Simpson**	**Chao1**	**ACE**
LN	754.9 ± 201.39	5.99 ± 0.69	0.9 ± 0.09	1,002.2 ± 233.4	1,056.1 ± 245.7
LOB	688.1 ± 185.6	5.3 ± 0.8^a^	0.9 ± 0.06 ^a^	935.2 ± 255.3	1,000.6 ± 267.0
HN	431.2 ± 26.9^a^	5.9 ± 0.4	0.9 ± 0.02	510.9 ± 50.0^a^	514.2 ± 45.0^a^
HOB	462.5 ± 71.9^c^	5.2 ± 0.4^b^	0.9 ± 0.03 ^b^	533.8 ± 85.9^c^	539.5 ± 85.2^c^
*p-*Value	0.000	0.000	0.003	0.000	0.000

LN, low-altitude obese children; LOB, low-altitude obese children; HN, high-altitude normal weight children; HOB, high-altitude obese children. Kruskal-Wallis test was used to test the significance of the OTUs, Shannon index, Simpson index, Chao 1, and abundance-based coverage estimator (ACE) index.

a Compared with LN group (p < 0.05).

b Compared with HN group (p < 0.05).

c Compared with LOB group (p < 0.05).

### β-Diversity analysis

**Figure 1:**
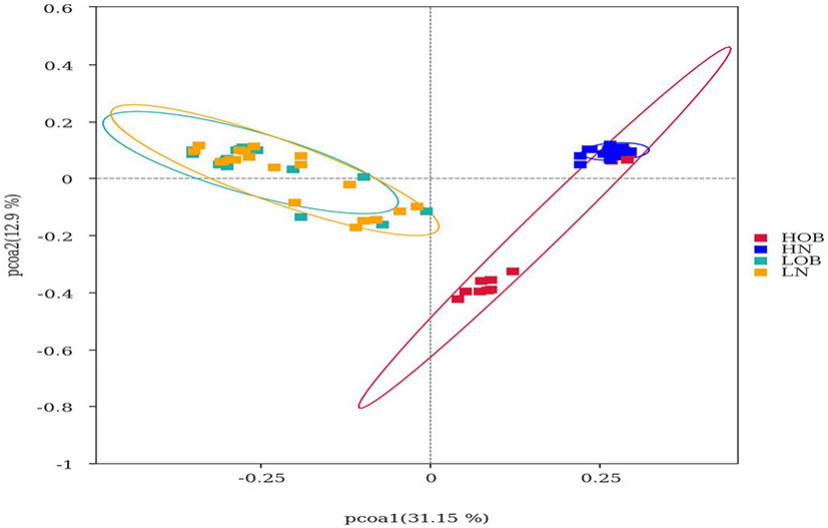
Beta diversity analysis based on UniFrac analysis. Red dot represents the high altitude obese children (HOB) group. Blue dot represents the high altitude normal weight children (HN) group. Green dot represents the low altitude obese children (LOB) group. Orange dot represents the low altitude normal weight children (LN) group. Circles in red, blue, orange, and green represent different periodontal bacterial community clusters, respectively. LN, low-altitude obese children; LOB, low-altitude obese children; HN, high-altitude normal weight children; HOB, high-altitude obese children.

β-diversity analysis was used to evaluate differences in species composition among the samples from different groups. Fecal microbial OTU data were examined by principal coordinate analysis (PCoA) to evaluate similarities among the four groups. Multiresponse permutation procedures (MRPPs) were used to analyse differences in microbial community structures between groups. The results showed that the groups from different altitudes had different microbiota compositions. The LN and LOB groups had similar microbiota compositions, and the sequences could be grouped into the same clusters, whereas the HN and HOB groups did not have similar microbiota compositions, and the sequences could not be grouped into the same clusters. In addition, the results revealed obvious differences in the community compositions and structures in the groups from different altitudes (Table 3 and Figure 1).

**Table 3:** MRPP analysis of differences in microbial community structure between groups.

**Group**	**A**	**Observed-delta**	**Expected-delta**	***p*-value**
LN-LOB	0.016	0.505	0.513	0.015
HN-HOB	0.048	0.556	0.584	0.001
LN-HN	0.050	0.486	0.511	0.001
LOB-HOB	0.073	0.588	0.634	0.001

Multiresponse permutation procedures (MRPP) analysis of differences in microbial community structure between group, A = 1 – (observed-delta/expected-delta), the smaller the Observe Delta value, the smaller the difference within the group, and the larger the Expect delta value, the larger the difference between the groups. A > 0, indicates that the difference between groups is greater than the difference within the group, A < 0, indicates that the difference within the group is greater than the difference between the groups.

LN, low-altitude obese children; LOB, low-altitude obese children; HN, high-altitude normal weight children; HOB, high-altitude obese children.

### Relative abundance analysis

**Figure 2:**
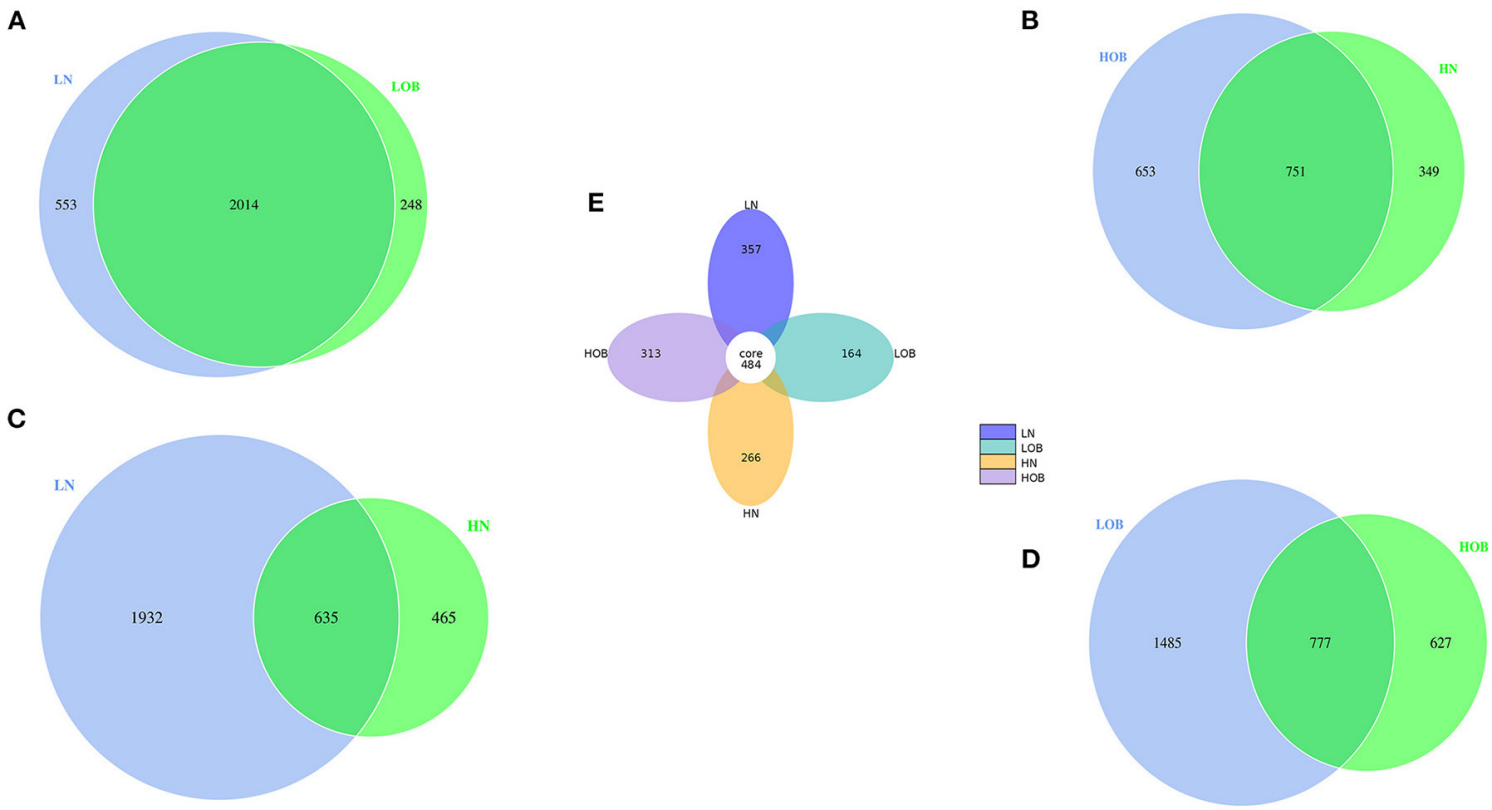
**(A–E)** The comparison between the two groups was done using the Venn chart and multiple groups using petal chart. Different colored circles represent different groups. Overlapping parts of the circles represent common OTUs between two groups, none overlapping parts represent unique OTUs. LN, low-altitude obese children; LOB, low-altitude obese children; HN, high-altitude normal weight children; HOB, high-altitude obese children.

**Figure 3:**
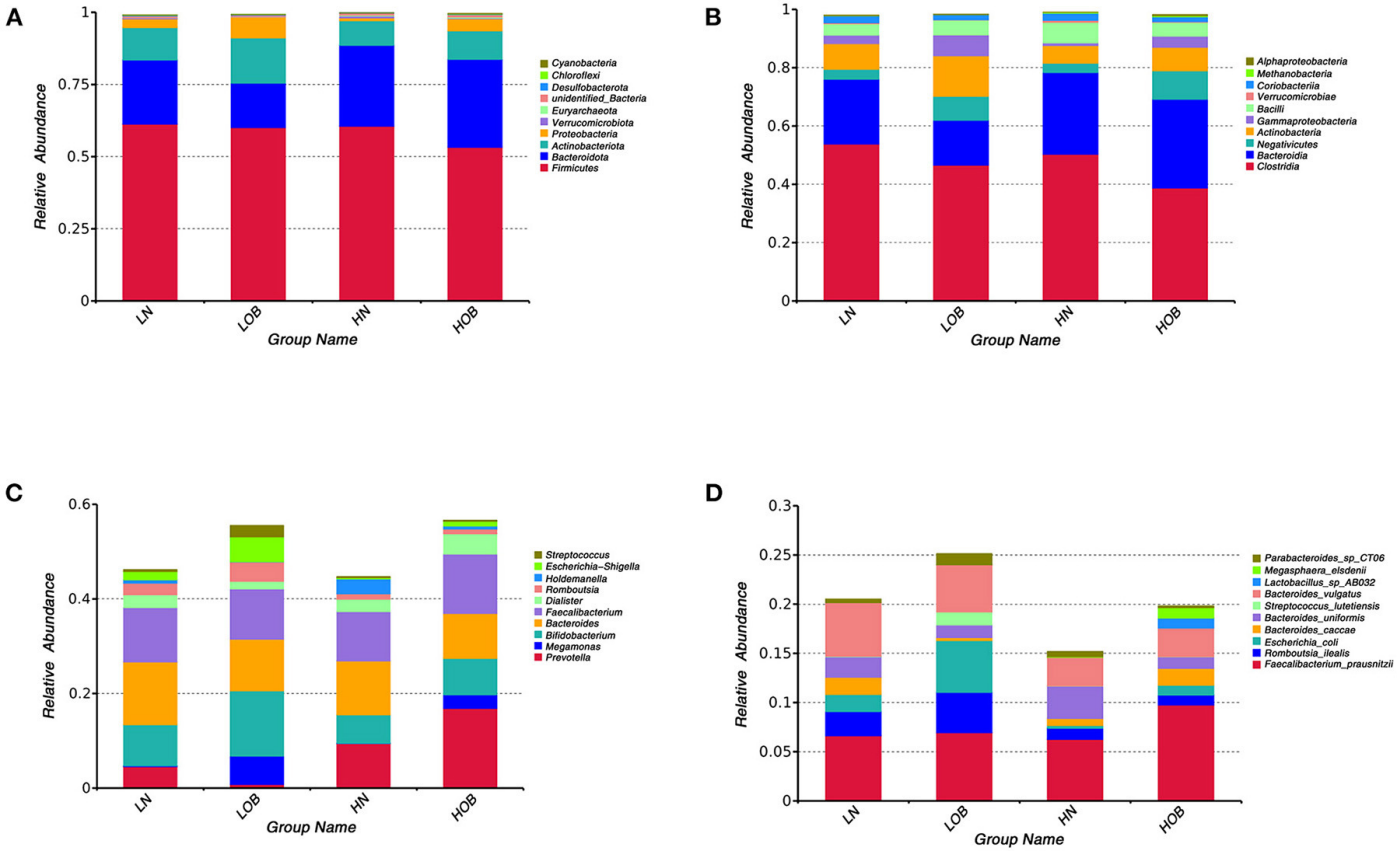
Community composition of fecal microbiota in four groups. In the bar chart, each bar represents the average relative abundance of each bacterial taxon. The taxa with high relative abundances at the phylum level (top 10, **A**), class level (top 10, **B**), genus level (top 10, **C**) and species level (top 10, **D**) are shown. LN, low-altitude obese children; LOB, low-altitude obese children; HN, high-altitude normal weight children; HOB, high-altitude obese children.

In this study, we found 484 common OTUs among the four groups, and there were 357, 164, 266, and 313 unique OTUs in the LN, LOB, HN, and HOB groups, respectively. The relative abundance of the microbiota was lowest in the LOB group (Figure 2). Further analysis of the microbiota at the phylum, class, genus and species levels in the four groups showed that five of the top 10 most dominant bacteria at any level were the same among the four groups. The microbiota compositions in all four groups were dominated by the following phyla (order: LN, LOB, HN, HOB): *Firmicutes* (61.2, 60.1, 60.6, and 53.2%, respectively); *Bacteroidota* (22.2, 15.4, 28.0, and 30.4%, respectively); *Actinobacteriota* (11.3, 15.7, 8.5, and 9.9%, respectively); *Proteobacteria* (3.0, 7.2, 0.9, and 4.2%, respectively); and *Verrucomicrobiota* (0.3, 0.05, 0.7, and 0.2%, respectively; Figure 3). At the phylum level, the relative abundance of *Firmicutes (F)* was higher than that of *Bacteroides (B)* in the obese groups. *Bacteroides* abundances and F/B ratios were significantly different among the four groups (Supplementary Table S1). At the phylum level, the abundances of *Bacteroidota, Proteobacteria*, and *Verrucomicrobiota* were significantly different among the four groups (Table 4). At the class level, the abundances of *Clostridia, Negativicutes, Gammaproteobacteria, Bacteroidia, Actinobacteria*, and *Verrucomicrobiae* were significantly different among the four groups (Table 4). At the genus level, the abundances of *Megamonas, Prevotella, Akkermansia*, and *Bifidobacterium* were significantly different among the four groups (Table 4). At the species level, the abundances of *Romboutsia, Bacteroides caccae*, and *Escherichia coli* were significantly different among the four groups (Table 4).

**Table 4:** Comparison of dominant OTUs in four groups at different level.

**Level**	**OTUs**	**LN**	**LOB**	**HN**	**HOB**	***p*-value**
Phylum	*Firmicutes*	61.2 ± 12.3	60.1 ± 9.3	60.5 ± 7.8	53.3 ± 11.9	0.120
	*Bacteroidota*	22.2 ± 13.9	15.4 ± 10.0	28.0 ± 9.7	30.4 ± 13.3	0.003
	*Actinobacteriota*	11.3 ± 8.9	15.7 ± 10.4	8.5 ± 5.0	9.9 ± 1.0	0.115
	*Proteobacteria*	3.3 ± 0.3	7.2 ± 0.8	1.0 ± 0.9	4.2 ± 0.4	0.000
	*Verrucomicrobiota*	0.3 ± 0.06	0.05 ± 0.001	0.7 ± 0.01	0.2 ± 0.01	0.002
Class	*Clostridia*	53.8 ± 13.5	46.6 ± 11.9	50.3 ± 9.2	38.7 ± 12.7	0.004
	*Bacteroidia*	22.2 ± 13.9	15.4 ± 10.0	28.0 ± 9.7	30.4 ± 13.3	0.003
	*Negativicutes*	3.4 ± 3.8	8.3 ± 13.8	3.2 ± 6.0	9.8 ± 13.1	0.021
	*Actinobacteria*	8.8 ± 9.0	13.9 ± 9.5	6.1 ± 4.0	8.1 ± 10.0	0.021
	*Gammaproteobacteria*	2.9 ± 3.3	7.1 ± 8.4	0.9 ± 1.1	3.8 ± 4.9	0.000
	*Verrucomicrobiae*	0.3 ± 0.6	0.1 ± 0.1	0.6 ± 0.2	0.2 ± 0.7	0.000
Genus	*Prevotella*	4.5 ± 13.4	0.8 ± 2.0	9.4 ± 11.2	16.8 ± 15.0	0.000
	*Megamonas*	0.2 ± 0.2	6.0 ± 14.4	0.02 ± 0.04	2.9 ± 6.0	0.000
	*Bifidobacterium*	8.7 ± 9.0	13.8 ± 9.5	6.0 ± 4.0	7.7 ± 10.1	0.015
	*Bacteroides*	13.3 ± 9.1	10.9 ± 7.1	11.4 ± 7.0	9.5 ± 10.4	0.284
	*Faecalibacterium*	11.5 ± 5.5	10.6 ± 6.3	10.4 ± 4.2	12.6 ± 8.9	0.942
	*Akkermansia*	0.3 ± 0.06	0.05 ± 0.001	0.7 ± 0.01	0.2 ± 0.01	0.002
	*Dialister(g)*	2.7 ± 0.4	1.6 ± 0.15	2.6 ± 0.59	4.3 ± 0.55	0.473
Species	*Faecalibacterium*	6.6 ± 3.7	6.9 ± 4.7	6.3 ± 2.8	9.7 ± 8.1	0.965
	*Romboutsia*	2.5 ± 1.4	4.1 ± 5.9	1.1 ± 0.8	1.0 ± 0.8	0.000
	*Escherichia-coli*	1.7 ± 2.2	5.3 ± 7.4	0.3 ± 0.2	1.0 ± 1.5	0.000
	*Bacteroides-caccae*	1.7 ± 3.8	0.3 ± 0.3	0.7 ± 0.5	1.7 ± 5.2	0.006
	*Bacteroides-uniformis*	2.1 ± 1.7	1.3 ± 1.6	3.3 ± 4.5	1.2 ± 1.9	0.096

LN, low-altitude obese children; LOB, low-altitude obese children; HN, high-altitude normal weight children; HOB, high-altitude obese children. Values are mean ± SD.

Kruskal–Wallis test was used to test the significance of different microbiota in four groups.

### Dominant gut microbiota composition between children living at different altitudes

**Figure 4:**
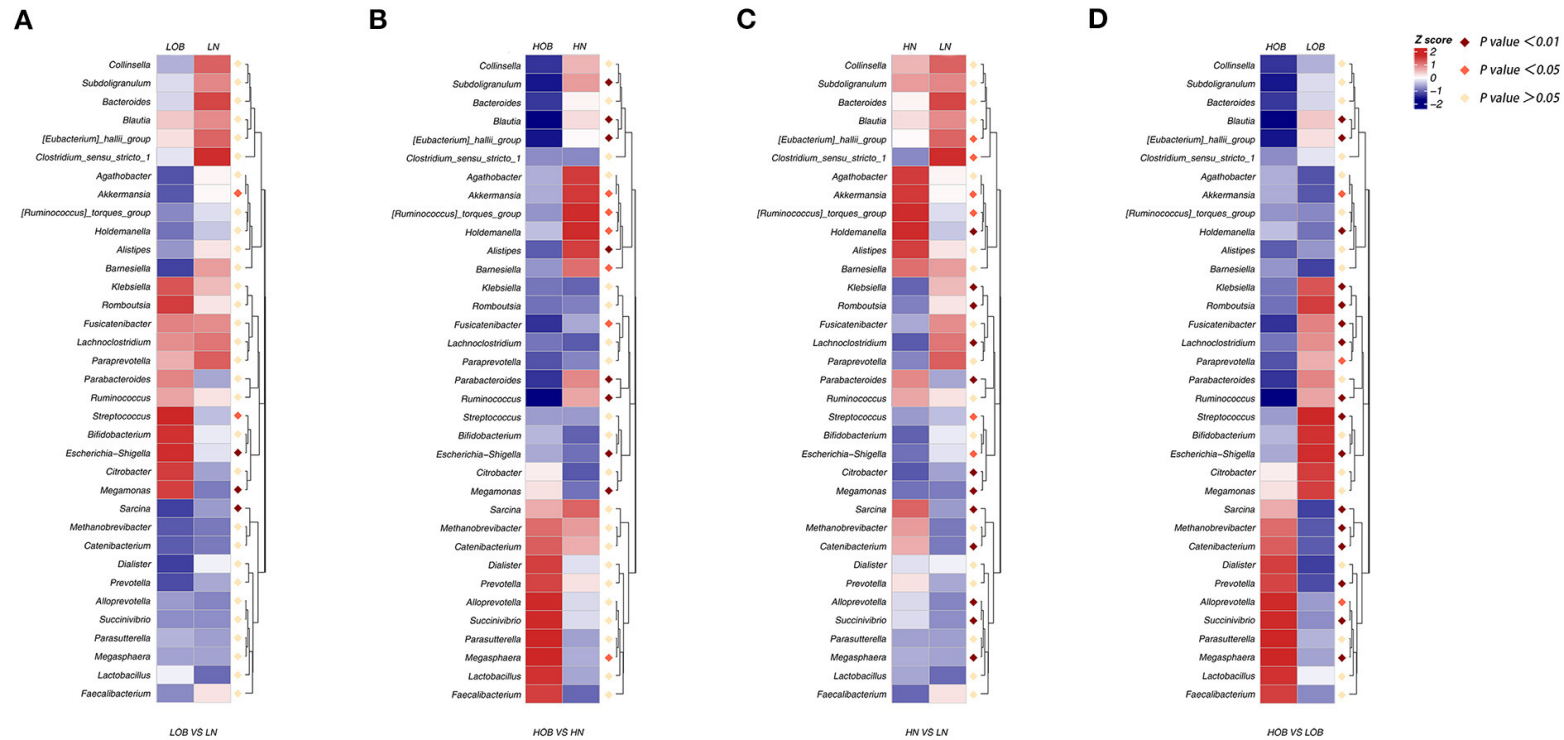
**(A–D)** MetaStats analysis, in which the abundances of the 35 most significantly different taxa in the two groups are shown in heatmap. Purple dot represents a high significant difference (*p* < 0.01), red dot represents a significant difference (*p* < 0.05), and yellow dot represents no significant difference (*p* > 0.05). LN, low-altitude normal weight children; LOB, low-altitude obese children; HN, high-altitude normal weight children; HOB, high-altitude obese children.

We further analyzed differences in the microbiota composition between the two different groups at the genus level using the Metastat analysis method. First, between groups from the same altitude, there were significant differences in the abundances of *Akkermansia, Streptococcus, Escherichia-Shigella, Megamonas* and *Sarcina* between the LN and LOB groups (*p* < 0.05; Figure 4A), and there were significant differences in the abundances of *Akkermansia, Ruminococcus torques, Holdemanella, Barnesiella, Fusicatenibacter, Megasphaera, Subdoligranulum, Blautia, Eubacterium hallii, Alistiples, Parabacteroides, Ruminococcus, Escherichia-Shigella* and *Megamonas* between the HN and HOB groups (*p* < 0.05; Figure 4B). Second, between groups with the same BMI, the abundances of *Eubacterium hallii, Clostridium sensu stricto, Akkermansia, Ruminococcus torques, Streptococcus, Escherichia-Shigella, Holdemanella, Klebsiella, Romboutsia, Lachnoclostridium, Parabacteroides, Citrobacter, Megamonas, Sarcina, Catenibacterium, Alloprevotella, Succinlvibrlo* and *Megasphaera* were significantly different between the HN and LN groups (*p* < 0.05; Figure 4C), and the abundances of *Blautia, Eubacterium hallii, Holdemanella, Klebsiella, Romboutsia, Fusicatenibacter, Lachnoclostridium, Ruminococcus, Streptococcus, Escherichia-Shigella, Sarcina, Methanobrevibacter, Catenibacterium, Prevotella, Succinlvibrlo, Megasphaera, Akkermansia, Paraprevotella and Alloprevotella* (*p* < 0.01) were significantly different between the HOB and LOB groups (*p* < 0.05; Figure 4D). In conclusion, we found that *Akkermansia, Holdemanella, Streptococcus, Prevotella, Escherichia-Shigella*, and *Megamonas* were strongly associated with BMI, and these six members of the microbiota plus *Sarcina* and *Catenibacterium* were associated with altitude.

### Differences in the gut microbiota composition between the different BMI groups

**Figure 5:**
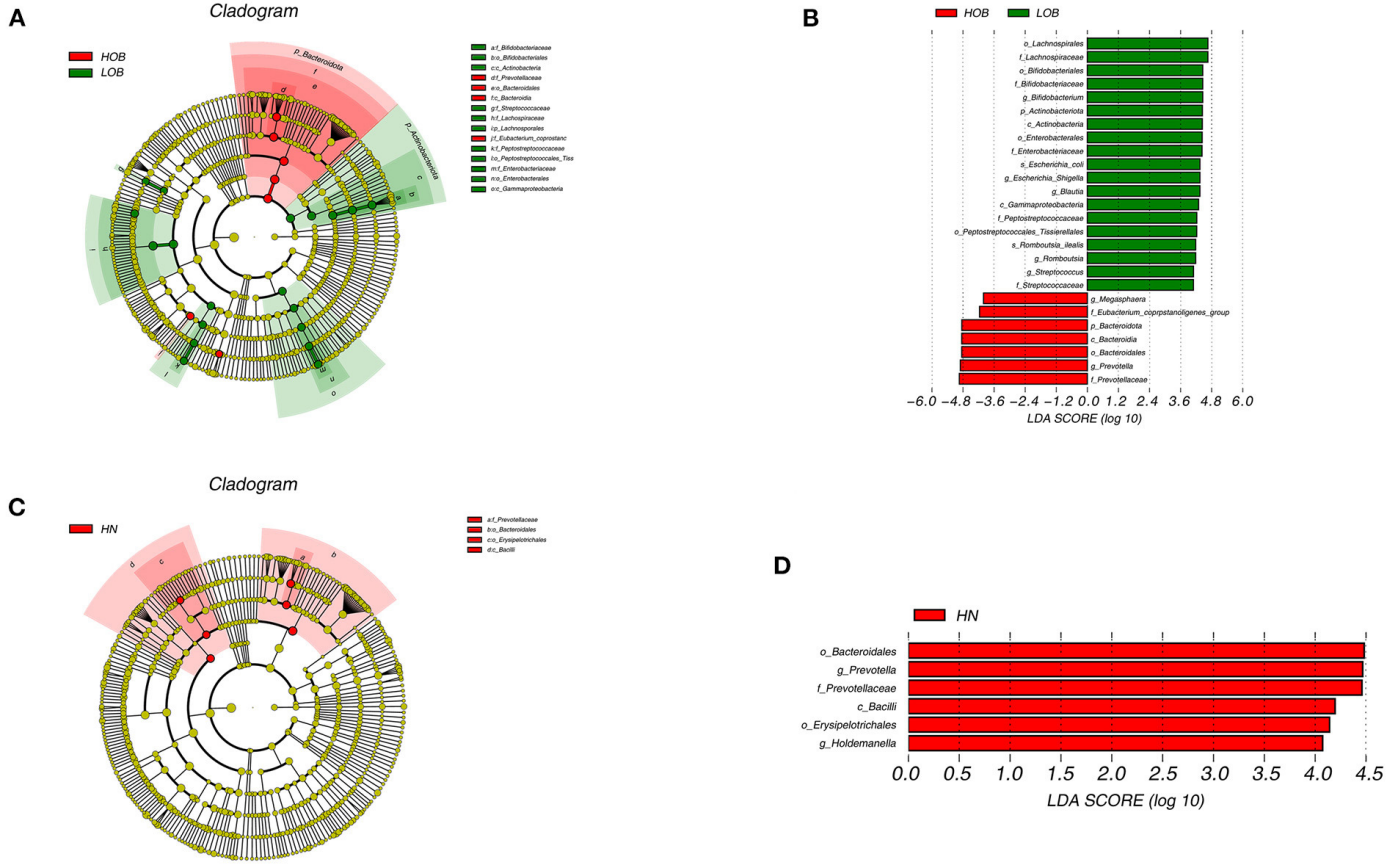
Cladogram based on LEfSe analysis showing the different abundance bacteria between two groups. Comparison between high-altitude obese children (HOB) and low-altitude obese children (LOB) **(A)**. Comparison between high-altitude normal weight children(HN) and low-altitude normal weight children (LN) **(C)**, with a linear discriminant analysis (LDA) threshold of 4 **(B,D)**. The bacteria with significantly different abundance at different taxonomic levels among groups were highlighted by colored circles and shadings.

LEfSe was performed to identify particular taxa with significantly different abundances between groups. The results of the UPGMA analysis showed that there were differences in the abundances of the genera *Bifidobacterium, Escherichia-Shigella, Blautia, Romboutsia, Streptococcaceae, Megasphaera*, and *Prevotella* in obese children living at different altitudes. *Bifidobacterium, Escherichia-Shigella, Blautia, Romboutsia, and Streptococcaceae* were highly abundant in children living at low altitudes, while *Megasphaera and Prevotella* were abundant in children living at high altitudes. These results indicated that these microbes were closely related to altitude when obesity conditions were the same. We further analyzed intestinal differences in normal-weight children living at different altitudes. *Prevotella* and *Holdemanella* were highly abundant at high altitudes. It was interesting to further confirm that *Prevotella* was positively correlated with high altitude (Figure 5).

### Correlations between the gut microbiota composition and BMI, TC, TG, HDL-C, and LDL-C levels

**Figure 6:**
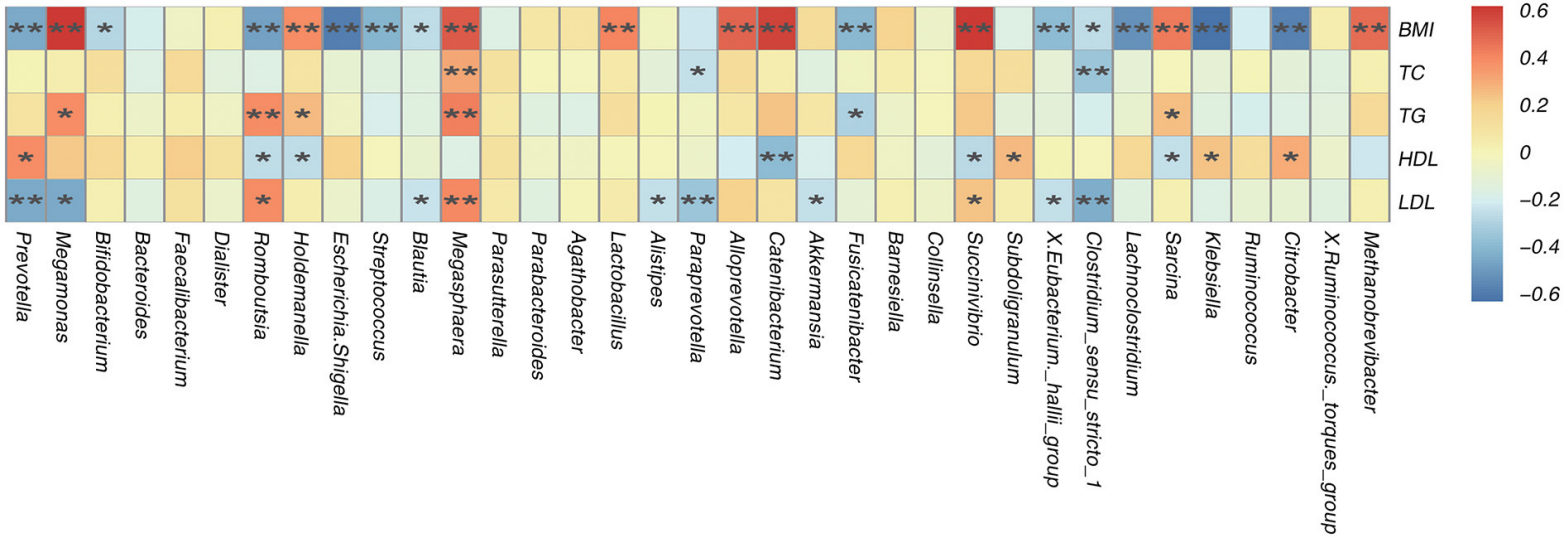
Spearman's association analysis of bacterial genera and BMI, TC, TG, HDL, and LDL. *r* indicates Spearman's correlation coefficient. Cells are colored based on the value of *r* between significantly altered genera and body mass index (BMI), total cholesterol (TC), tri glyceride (TG), high-density lipoprotein cholesterol (HDL-C), and low-density lipoprotein cholesterol (LDL-C). Red represents a significantly positive correlation, blue represents a significant negative correlation, and yellow represents no significant correlation. ^*^p < 0.05; ^**^p < 0.01.

A Spearman's correlation matrix was generated to analyse correlations between BMI, TC, TG, HDL-C, and LDL-C levels and the dominant genera in the gut microbiota in all children. Significant relationships were observed between the gut microbiota composition and BMI, TC, TG, and LDL-C levels. Correlation analysis revealed that the abundances of the genera *Methanobrevibac, Sarcina, Succinivibrio, Catenibacterium, Alloprevotella, Lactobacillus, Megasphaera, Holdemanella* and *Prevotella* were positively correlated with BMI (*p* < 0.05; Figure 6), and the abundances of *Citrobacter, Klebsiella, Lachnoclostridum, Clostridium-sens, X. Eubacterium.-h, Fusicatenibacter, Blautia, Streptococcus, Escherichia. Shige, Romboutsia, Bifidobacterium* and *Megamonas* were negatively correlated with BMI (*p* < 0.05; Figure 6). The abundance of *Megasphaera* was positively correlated with the TC level (*p* < 0.05; Figure 6), and the abundances of *Clostridium and Paraprevotella* were negatively correlated with the TC level (*p* < 0.05; Figure 6). The abundances of *Sarcina, Megasphaera* and *Holdemanella* were positively correlated with the TG level (*p* < 0.05; Figure 6), and the abundances of *Fusicatenibacter* and *Romboutsia* were negatively correlated with the TG level (*p* < 0.05; Figure 6). The abundances of *Citrobacter, Klebsiella, Subdoligranulum* and *Romboutsia* were positively correlated with the HDL-C level (*p* < 0.05; Figure 6), and the abundances of *Sarcina, Scuccinivibrio, Catenibacterium, Holdemanella* and *Prevotella* were negatively correlated with the HDL-C level (*p* < 0.05; Figure 6). The abundances of *Succinivibrio* and *Megasphaera* were positively correlated with the LDL-C level (*p* < 0.05; Figure 6), and the abundances of *Clostridium_sens, X. eubacterium._h, Akkermansia, Paraprevotella, Alistipes, Blautia*, and *Romboutsia* were negatively correlated with the LDL-C level (*p* < 0.05; Figure 6). In addition, the difference in the abundance of *Prevotella* was positively correlated with the difference in the LDL level between the obese groups from different altitudes (Figure 6).

### Functional prediction of gut microbiota in obese Tibetan children living at different altitudes

**Figure 7:**
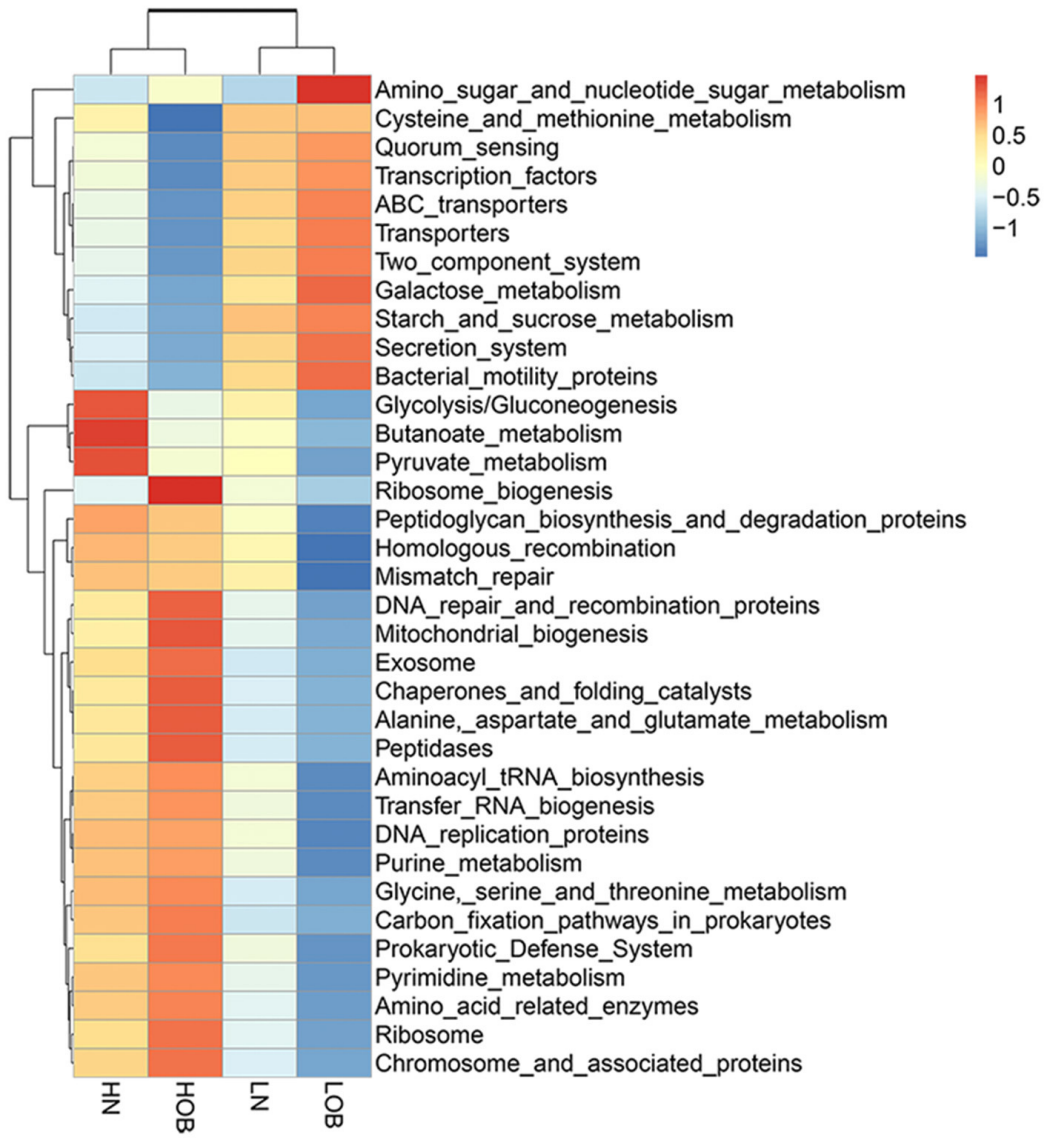
Functional profiles of the fecal microbial communities in four groups. The 35 most-abundant pathways at the third level of KEGG pathways are shown in the heatmap. LN, low-altitude normal weight children; LOB, low-altitude obese children; HN, high-altitude normal weight children; HOB, high-altitude obese children.

To further explore the function of gut microbes in children at different altitudes, Tax4Fun was used to analyse the functional prediction of the fecal microbiota in different groups. Figure 7 shows the 35 most abundant pathways at the third level of KEGG pathways. Of these 35 pathways, 10 pathways (amino sugar and nucleotide sugar metabolism; quorum sensing; transcription factors; ABC transporters; transporters; two component system; starch and sucrose metabolism; galactose metabolism; secretion system, bacterial motility proteins.) were enriched in the fecal microbial community of the LOB groups. Eighteen pathways (ribosome biogenesis; prokaryotic defense system; aminoacyl tRNA biosynthesis; transfer RNA biogenesis; DNA replication proteins; purine metabolism; glycine, serine and threonine metabolism; carbon fixation pathways in prokaryotes; pyrimidine metabolism; amino acid-related enzymes; ribosome; chromosome and associated proteins; DNA repair and recombination proteins; mitochondrial biogenesis; exosome; chaperones and folding catalysts; alanine, aspartate and glutamate metabolism; peptidases.) were enriched in the HOB groups, and three pathways (glycolysis/gluconeogenesis, butanoate metabolism and pyruvate metabolism) were enriched in the HN groups. The KEGG pathways for predicting the function of gut microbiota were mainly expressed in four aspects: metabolism, genetic information processing, environmental information processing and cellular processes. The differences in these pathways may be related to the influence of altitude on the abundance of intestinal flora. In addition, the prominence of the butyric acid metabolic pathway in normal weight children at high altitude has attracted our attention.

### Correlation analysis between KEGG pathways and blood lipids

Through KEGG pathway annotation and enrichment analysis, the results showed that the butyric metabolism pathway was significantly different in the four groups, with the highest abundance in the HN group, followed by the LN group, and the lowest abundance in the LOB group (Figure 7). Combined with this feature, we measured the short-chain fatty acids in the feces of different groups, and the results showed that acetic, propionic and butyric acid levels were significantly different among the four groups (Table 5). We further carried out a correlation analysis between blood lipids and the content of short-chain fatty acids, and the results showed that acetic acid was correlated with LDL, while butyric acid was correlated with TC, TG, and LDL (Table 6).

**Table 5:** Comparison of SCFAs in the four groups.

**SCFAs**	**LN**	**LOB**	**HN**	**HOB**	**F**	***p*-value**
Acetate	1,919.5 ± 76.5^b^^c^^d^	2,008.9 ± 61.7^a^^b^^d^	2,121.70 ± 102.6^a^^c^^d^	2,432.2 ± 88.5^a^^b^^c^	113.10	0.000
Propionate	1,517.5 ± 274.5^c^^d^	1,181.8 ± 46.7^a^^b^	1,428.2 ± 239.9^c^	1,332.0 ± 252.7^a^	6.69	0.001
Butyrate	1,175.3 ± 67.6^b^^c^^d^	1,250.9 ± 123.2^a^^b^^c^^d^	1,528.8 ± 82.4^a^	1,516.7 ± 87.8^a^	73.70	0.000

LN, low-altitude obese children; LOB, low-altitude obese children; HN, high-altitude normal weight children; HOB, high-altitude obese children.

Values are mean ± SD. One-way ANOVA test was used to test the significance of different microbiota in four groups.

a Compared with LN group (*p* < 0.05).

b Compared with HN group (*p* < 0.05).

c Compared with LOB group (*p* < 0.05).

d Compared with HOB group (*p* < 0.05).

**Table 6:** Correlation between SCFAs and blood lipids.

**SCFAs**	**TC**		**TG**		**LDL**		**HDL**	
	***r***	***p*-value**	***r***	***p*-value**	***r***	***p*-value**	***r***	***p*-value**
Acetate	0.109	0.369	0.021	0.865	0.350	0.003	−0.124	0.306
Propionate	−0.099	0.414	−0.167	0.167	−0.124	0.306	0.205	0.089
Butyrate	0.269	0.025	−0.292	0.011	0.361	0.002	0.026	0.831

## Discussion

Obesity is linked to many chronic diseases (2–5). The steadily increasing prevalence of excess body weight among children and adolescents is currently one of the greatest challenges for public health authorities worldwide (40–42). Many studies have shown that obesity in childhood is associated with an increased risk of obesity in adulthood. This situation reminds us to pay attention to the problem of childhood obesity. The potential link between obesity and the composition of the gut microbiota has attracted the attention of many researchers. Many studies have shown that disruption of the gut microbiota composition may be an important cause of obesity (16, 43, 44). However, the gut microbiota is also affected by altitude, and the low prevalence rate of obesity in high-altitude areas is of interest (45, 46). Short or chronic exposure of humans or animals to hypoxic conditions (the most typical characteristic of high altitude) can affect the composition of the intestinal microbiota (47–50). In this study, we focused on the composition, structure and diversity of the gut microbiota in obese Tibetan children aged 7–12 years who lived at different altitudes. By exploring the intestinal bacteria that affect weight change in high-altitude areas, we have provided evidence to guide early obesity prevention measures in the future. In addition, we also analyzed correlations between the gut microbiota composition and lipid metabolism indicators.

Obesity is a health problem among children that contributes to the occurrence of lipid disorders and abnormal blood pressure. It is often accompanied by increases in TC, TG, and LDL-C levels and decreases in HDL-C levels (51). We obtained similar results in our study. Obesity can also cause changes in the gut microbiota composition. In recent years, changes in bacterial strains in the human intestine have been proposed to play a causative role in obesity (52–54). Research by Ley et al. (55) showed that obesity affects the diversity of the gut microbiota, and the relative proportion of *Bacteroidetes* is decreased in obese people compared with that in lean people (16). These authors also obtained the same results in mice (55), and we drew the same conclusion. We found that at the phylum level, the relative abundance of *Firmicutes (F)* was higher than that of *Bacteroides (B)* in the obese groups. The F/B ratios were significantly different between the obese groups. *Megamonas* is a genus of *Firmicutes*. In our study, *Megamonas* was present in only the obese groups, and the relative abundance in the LOB group was higher than that in the HOB group and was positively correlated with BMI and negatively correlated with the LDL-C level. A study conducted in Nanjing (sea level), China, showed that height, weight, age, BMI, TG levels and creatinine levels in children were positively correlated with the relative abundance of *Megamonas* and that TC, HDL-C and LDL-C levels were negatively correlated with the relative abundance of *Megamonas* (56). Otoniel Maya-Lucas' research also showed that the relative abundance of *Megamonas* in obese Mexican people living at an altitude of 2,200 m was correlated with height, weight and BMI (57). Therefore, we concluded that *Megamonas* is associated with obesity. In addition, it has been reported that *Megamonas* can ferment glucose to form acetate and propionate, which have been shown to be substrates for lipogenesis and cholesterol formation as energy sources for the host (58). This further explains the high relative abundance of *Megamonas* in obese subjects. These findings are consistent with our findings. The results of Ma (59) showed that the relative abundance of *Megamonas* in people in the plains area was higher than that in people in the high-altitude area, consistent with the conclusion of our study. The reason may be related to the impairment of gastrointestinal mucosal barrier function caused by hypoxia, but research on the mechanism is still incomplete.

The gut microbiota is affected by altitude. A high-altitude environment is characterized by low pressure, hypoxia, strong radiation, cold temperatures, etc. Hypoxia can affect the behavior and activities of human beings (60). Hypoxia may also alter the composition of the gut microflora (61), and substantial evidence has implicated both aerobic and facultative anaerobic intestinal bacteria in the dynamic configuration and stability of the anaerobic environment inside the gut (62, 63). Li et al. (25) compared the gut microbiomes of people living at different altitudes and found that the composition of the gut microbiota in individuals living at high altitudes was lower than that in individuals living on the plains. In our study, we found that the abundances of *Akkermansia, Holdemanella, Streptococcus, Prevotella, Escherichia-Shigella, Megamonas, Sarcina* and *Catenibacterium* were associated with altitude. *Akkermansia, Holdemanella, Prevotella, Sarcina*, and *Catenibacterium* were highly abundant in those living at high altitudes, while *Streptococcus, Escherichia-Shigella*, and *Megamonas* were present at significant levels in those living at low altitudes. Of these, *Prevotella* deserves additional attention. The relative abundance of *Prevotella* in the high-altitude groups was significantly higher than that in the low-altitude groups. However, when comparing the groups with different BMIs at the same altitudes, the abundance of *Prevotella* was higher in the HOB group than in the HN group, while the opposite was observed between the low-altitude groups. This study has shown (64) that the Tibetan microbiome is characterized by a relative abundance of *Prevotella* in individuals living at high altitudes (3,600 m). This difference is likely related to dietary structure. The distribution and prevalence of *Prevotella* in the human gut is influenced by a variety of factors, including body condition, lifestyle, sex and age (65). Some scholars (66) have stated that the abundance of *Prevotella* is associated with the consumption of a diet rich in carbohydrates. The Qinghai-Tibet Plateau is rich in highland barley (a high-fiber wheat), which is the main source of carbohydrates among local people. Other studies have shown (67) that the abundance of *Prevotella* is positively correlated with the consumption of high-fiber foods. However, in our study, to focus on the effect of hypoxia on the gut microbiome, we standardized the diets of the study subjects. High-altitude environments may place greater energy demands on mammals due to thermoregulatory stress than low-altitude environments (68–70). Many anaerobic gut bacteria produce short-chain fatty acids (SCFAs) as end products of polysaccharide fermentation (71). A greater abundance of SCFA-producing obligate anaerobes has been reported in populations who live at high altitudes (25, 64, 72–74) A hypoxic environment leads to the enrichment of facultative anaerobic bacteria (*Prevotella*) that produce SCFAs and enhances the ability of intestinal bacteria to produce SCFAs to provide more energy, regulate blood pressure, and maintain the stability of the intestinal environment in people who live in high-altitude regions (75). The relative abundance of *Prevotella* increases with altitude, and this change could be explained by differences in diet, climate, atmospheric oxygen, or any other variable correlated with altitude. Moreno's study (76) provided strong evidence that reduced atmospheric oxygen alone caused an increase in *Prevotella* abundance. A previous study found that high-altitude exposure altered the gastrointestinal system, causing pathological conditions such as upper gastrointestinal bleeding, ulcers, vomiting, diarrhea, and anorexia. This may be the cause of hypobaric hypoxia-induced gut microbial dysbiosis, which can be ameliorated by prebiotic/probiotic treatment (77). Another study explored the relationship between the hypothalamic-pituitary-thyroid (HPT) axis and gut microbiota composition under hypoxic conditions by simulating the effects of 5,500 m altitude on the HPT axis and gut microbiota in rats. The study found that compared with those in the normoxia group, there were significant differences in the relative abundances of 12 genera in the chronic hypoxia exposure group; thyrotropin-releasing hormone (TRH) and thyroid-stimulating hormone (TSH) concentrations were significantly lower in the hypoxia group than in the control group, and TT4 and TT3 concentrations were significantly higher in the hypoxia group than in the control group. There were significant correlations between the differential bacteria and HPT axis hormones in serum, and *Prevotella* was significantly negatively correlated with TSH (78). These results provide evidence that the increase in *Prevotella* abundance at high altitudes may be driven by lower atmospheric oxygen levels. Previous studies have shown that the biodiversity and abundance of the microbiota in obese individuals often differ from those in normal individuals. Our study found that the relative abundance of *Prevotella* in the obese group was lower than that in the normal group on the plain, but the opposite was true at high altitudes. Duan et al. (79) used 16S rRNA sequencing to compare the intestinal flora compositions of 21 obese individuals from Shandong Province (China) and 21 normal individuals from Beijing to study the characteristics of the intestinal flora in the obese population. The team found that at the species level, there were significant differences in nine species between the control and obese groups. The abundance of *Prevotella* was significantly increased in the obese population. This was consistent with our results in the groups at high altitudes. Barczyńska R studied the relationship between intestinal bacterial composition and weight in 20 obese children and 20 normal weight children and found that the *Prevotella* abundance was lower in the fecal microflora of obese children, with an average 30% higher abundance of *Prevotella* in normal weight children than in obese children. However, the difference was significant in only obese children and not in overweight children (80). Fernández-Navarro et al. (81) analyzed the interrelationships among obesity, diet, oxidative stress, inflammation and the intestinal flora composition in 68 healthy adults and found that the lower abundance of *Prevotella* in the obese group was associated with elevated proinflammatory and pro-oxidative states. Many other studies have also confirmed the effect of obesity on the intestinal flora composition and the low abundance of *Prevotella* in obese individuals (82–84). In our study, we found that the abundance of *Escherichia-Shigella* was also related to altitude. A comparative analysis of the four groups revealed that *Escherichia-Shigella* exhibited the lowest relative abundance in the HN group and the highest relative abundance in the LOB group, and the difference was statistically significant. *Escherichia-Shigella* is a member of the gram-negative Enterobacteriaceae family and causes enterobacteriosis, usually resulting in diarrhea and dysentery (85). *Escherichia*-*Shigella* is transmitted *via* the fecal-oral route (86), and poor water supply, lack of basic sanitation and unhygienic behavior have all been associated with *Escherichia-Shigella* infection. The low economic level and harsh climatic conditions of the Tibetan Plateau result in conditions that are favorable for the spread of *Escherichia-Shigella*. However, our results showed a low relative abundance of *Escherichia-Shigella* at high altitudes. Successful invasion of *Escherichia-Shigella* requires overcoming two gut-specific barriers: the microbiota and the mucus layer. Related studies have suggested that a *Prevotella*-rich microbiota may have a protective effect against *Escherichia-Shigella* infection (87). This explains the low *Escherichia-Shigella* abundance in high-altitude populations. In addition, the higher the inflammation score is, the higher the *Escherichia-Shigella* abundance (88). In a mouse study, *Escherichia-Shigella* abundance was positively correlated with blood lipid, glucose and insulin levels (89). This finding indicates that in the HOB group, a greater abundance of *Prevotella* reduces the inflammatory response caused by obesity. All of these results suggest that the *Escherichia-Shigella* abundance is higher in obese people than in normal-weight people. Therefore, we believe that the reason for the low prevalence of obesity in high-altitude areas may be related to the abundance of *Prevotella*.

Finally, we found a correlation between the gut microbe composition and blood lipid levels. Our results showed that the abundances of *Sarcina, Megasphaera* and *Holdemanella* were positively correlated with the TG level, while the abundances of *Fusicatenibacter* and *Romboutsia* were negatively correlated with the TG level. The abundances of *Succinivibrio* and *Megasphaera* were positively correlated with the LDL-C level, while the abundances of *Clostridium_sens, X. eubacterium._h, Akkermansia, Paraprevotella, Alistipes, Blautia* and *Romboutsia* were negatively correlated with the LDL-C level. Fu et al. (90) showed that the abundances of *Bacteroides, Akkermansia, Desulfovibrio* and *Parabacteroides* were negatively correlated with GLU, TG, TC and HDL-C levels and positively correlated with the LDL-C level. The abundance of *Ruminiclostridium* was positively correlated with GLU, TG, TC and HDL-C levels but negatively correlated with the LDL-C level. Our results are somewhat similar. In this study, we found that although obese children living at a high altitude also had an abnormal BMI and LDL-C level, their blood lipid levels were lower than those in children living at a low altitude. What is the relationship between blood lipid levels and gut microbe composition in populations who live at high altitudes? In our study, we found that the abundance of *Faecalibacterium* was higher in the high-altitude groups, especially in the HOB group. Faecalibacterium belongs to *Firmicutes*, which is fermented and metabolism to produce butyric acid in the intestine. Butyrate provides energy for the body through fatty acid oxidation (91). In this study, we conducted a study on the correlation between butyrate and blood lipids and found that butyrate was correlated with TC, TG, and LDL and negatively correlated with TC, indicating that butyrate has a certain regulatory effect on blood lipids. A study has shown that butyrate is involved in diet-induced obesity and insulin resistance (92) by downregulating the expression and activity of PPAR-γ, promoting a change from lipogenesis to lipid oxidation (93). This is consistent with our results. At the same time, we found that the butyric acid metabolic pathway has a high abundance in normal weight children through the KEGG metabolic pathway and the highest abundance in normal weight children who live in high altitude. Therefore, we inferred that altitude changed the abundance of *Faecalibacterium*, which further affected the production of butyrate.

## Conclusions

In this study, we found that the TG and LDL-C levels in the obesity groups were higher than those in the normal-weight groups, and those in the high-altitude obesity groups were lower than those in the low-altitude obesity groups. Altitude affects the composition and relative abundance of the gut microbiota. Groups living at different altitudes and with different body weights have their own dominant bacterial genera. *Megamonas* was closely related to obesity, and *Prevotella* was associated with altitude. *Prevotella* had an inhibitory effect on the abundance of *Escherichia-Shigella*. There were correlations between the gut microbiota composition and lipid metabolism indicators, and short-chain fatty acids play an important role in lipid regulation.

## Data availability statement

The datasets presented in this study can be found in online repositories. The names of the repository/repositories and accession number(s) can be found in the article/Supplementary material.

## Ethics statement

The studies involving human participants were reviewed and approved by the Ethics Committee of Qing Hai Provincial People's Hospital, Xining, China. Written informed consent to participate in this study was provided by the participants' legal guardian/next of kin. Written informed consent was obtained from the individual(s), and minor(s)' legal guardian/next of kin, for the publication of any potentially identifiable images or data included in this article.

## Author contributions

WD and WZ designed the experiments. YM, RJ, YH, and QZ performed the experiments. WD, ZW, XY, and AA analyzed the data. WD wrote the manuscript. LL provided financial support. All authors contributed to the article and approved the submitted version.

## Funding

This research was supported by Project of the Basic Research Program of Qinghai Provincial Science and Technology Department Fund (Gran Number: 2018-ZJ-758), High-end Innovative Talents Thousand Talents Program in Qinghai Province, and Leading Talents and Innovation Team Project Funding.

## Conflict of interest

The authors declare that the research was conducted in the absence of any commercial or financial relationships that could be construed as a potential conflict of interest.

## Publisher's note

All claims expressed in this article are solely those of the authors and do not necessarily represent those of their affiliated organizations, or those of the publisher, the editors and the reviewers. Any product that may be evaluated in this article, or claim that may be made by its manufacturer, is not guaranteed or endorsed by the publisher.
