# Supplementary figures and images for: Analysis of the gut microbiome in obese native Tibetan children living at different altitudes: A case–control study
Source: Front Public Health. 2022 Nov 24;10:963202. doi: 10.3389/fpubh.2022.963202 (PMC9731119; doi:10.3389/fpubh.2022.963202)

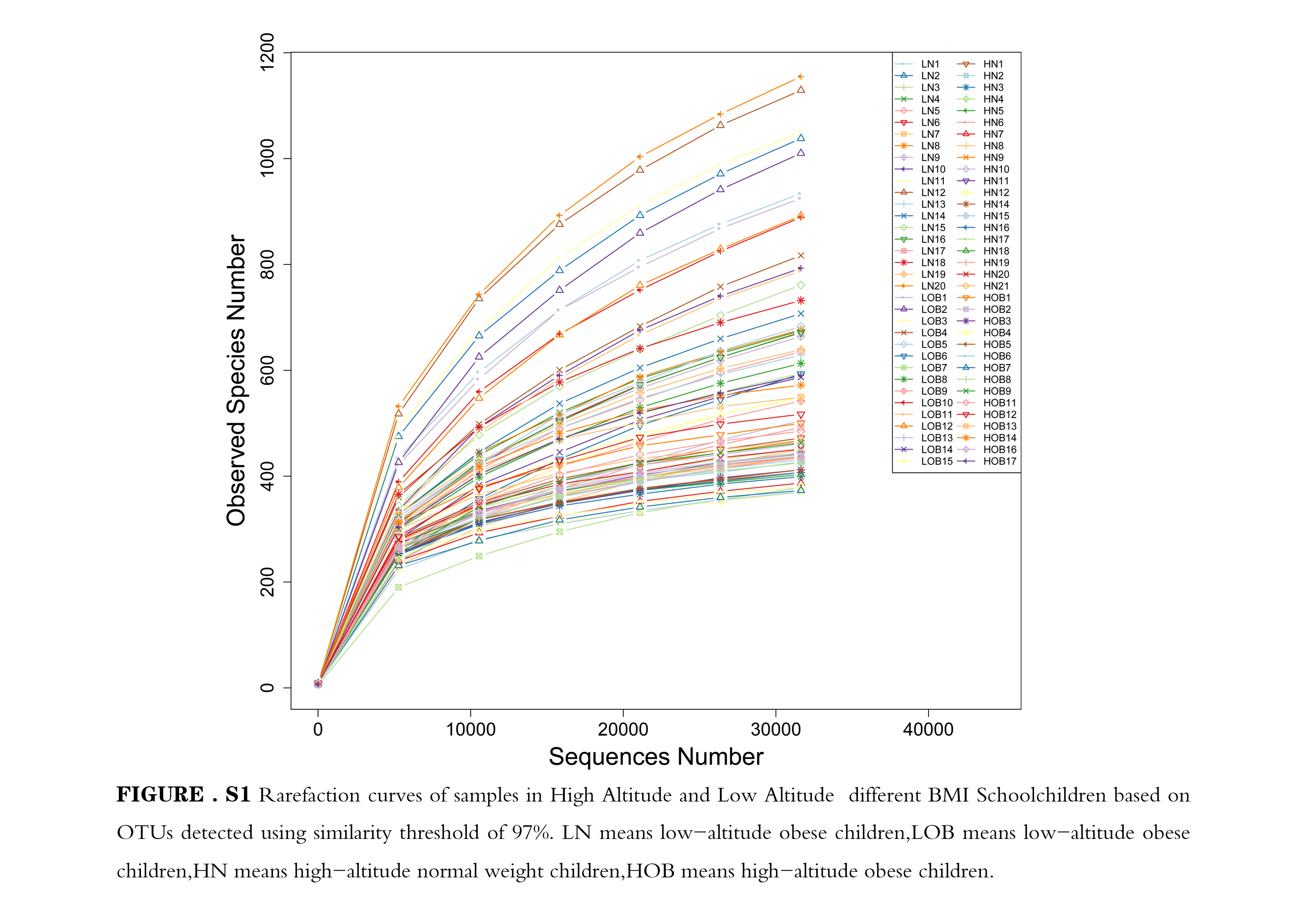

Supplement: Supplementary file 2 [file Image_1.TIF]
